# Modulation of Allergic Inflammation in the Nasal Mucosa of Allergic Rhinitis Sufferers With Topical Pharmaceutical Agents

**DOI:** 10.3389/fphar.2019.00294

**Published:** 2019-03-29

**Authors:** Annabelle M. Watts, Allan W. Cripps, Nicholas P. West, Amanda J. Cox

**Affiliations:** ^1^Menzies Health Institute Queensland, School of Medical Science, Griffith University, Southport, QLD, Australia; ^2^Menzies Health Institute Queensland, School of Medicine, Griffith University, Southport, QLD, Australia

**Keywords:** allergic rhinitis, intranasal, antihistamines, steroids, decongestants, anticholinergic, chromones

## Abstract

Allergic rhinitis (AR) is a chronic upper respiratory disease estimated to affect between 10 and 40% of the worldwide population. The mechanisms underlying AR are highly complex and involve multiple immune cells, mediators, and cytokines. As such, the development of a single drug to treat allergic inflammation and/or symptoms is confounded by the complexity of the disease pathophysiology. Complete avoidance of allergens that trigger AR symptoms is not possible and without a cure, the available therapeutic options are typically focused on achieving symptomatic relief. Topical therapies offer many advantages over oral therapies, such as delivering greater concentrations of drugs to the receptor sites at the source of the allergic inflammation and the reduced risk of systemic side effects. This review describes the complex pathophysiology of AR and identifies the mechanism(s) of action of topical treatments including antihistamines, steroids, anticholinergics, decongestants and chromones in relation to AR pathophysiology. Following the literature review a discussion on the future therapeutic strategies for AR treatment is provided.

## Introduction

Allergic rhinitis (AR) is estimated to affect between 10 and 40% of the population worldwide (Bjorksten et al., 2008; Bernstein et al., 2016) and is associated with significant medical and economic burden (Cook et al., 2007; Zuberbier et al., 2014; Marcellusi et al., 2015). AR is classified as a chronic upper respiratory disease whereby exposure to allergens induces an IgE mediated inflammation of the mucous membranes lining the nose (Bousquet et al., 2008). The disease manifests symptomatically as nasal congestion, rhinorrhoea, itchy nose and sneezing. Symptoms of post nasal drip, itchy/red eyes also occur in some sufferers. House dust mites, animals, and mold spores are major triggers responsible for perennial presentation of symptoms while exposure to pollen triggers seasonal symptoms (Cook et al., 2007). Complete avoidance of airborne allergens is not possible and without a cure, the available therapeutic options are typically focused on achieving symptomatic relief.

The nasal mucosa is the primary site for allergen exposure and the inflammatory reactions that cause AR symptoms. The mechanisms driving AR pathophysiology are multifaceted and include activation and migration of effector cells, release of mediators, chemokines and cytokines from inflammatory cells, and damage to the nasal epithelium and nerve endings. Oral (systemic) therapies, such as antihistamines, are commonly used to treat AR symptoms. However, topical therapies offer many advantages over oral therapies and are being continuously developed to target AR symptoms. Topical therapies allow for higher concentrations of drugs to be applied directly to the receptor sites at the source of inflammation (nasal mucosa) and carry a reduced risk of systemic side effects compared to oral therapies. Current therapies target different components of the allergic response, and consequently do not always offer full coverage of symptoms. Given the numerous immune cells, signaling molecules and mediators involved in the allergic response, development of a single therapy to rapidly target all components of the allergic response represents a significant challenge as a treatment option.

This review will: (i) consider the immune cells, mediators and messenger molecules of the allergic response, (ii) outline the time course of the allergic response, (iii) identify the mechanism for each topical drug and will indicate which components of the allergic response are modulated by the drug mechanism, and (iv) highlight the gaps in current therapy and identify future therapeutic strategies for the treatment of AR.

## Pathophysiology of Allergic Rhinitis

Atopy occurs as a result of a genetic predisposition to produce IgE antibodies and consequently the development of allergic disease. The IgE antibody is a fundamental component of the T-helper 2 (Th2) arm of the immune system, which exists as a means for defending the human body against helminth infection or other multi-cellular parasites (Allen and Sutherland, 2014). In atopic subjects, the Th2 immune pathway is instead promoted to produce an immune response to allergenic proteins derived from animals, molds and plant pollens. The allergenic proteins are processed by specialized cells of the immune system at mucosal barriers of the nose, resulting in the production of IgE antibodies. These newly produced IgE antibodies interact with specific allergens and immune cells (mast cells and basophils) situated in the nasal mucosa. The interaction of these antibodies, allergens and specialized cells, sets off a series of reactions whereby the resident mucosal immune cells such as mast cells, eosinophils and basophils to release powerful mediators such as histamine as well as chemokines, cytokines and adhesion molecules that encourage increased production of leukocytes in the bone marrow as well as attracting circulating effector leukocytes including neutrophils, Th2 lymphocytes, basophils and eosinophils into the nasal epithelium. In a series of time-dependent phases including sensitisation, early- and late-phase responses, these effector cell types, mediators and cell signaling molecules work in a complex network of interactions resulting in specific symptoms and the inflammatory morphology of AR (Bousquet et al., 2001).

### Antigen Presentation and Sensitisation

Antigen presenting cells (APCs) are located in para- and inter-cellular channels neighboring the basal epithelial cells in the nasal mucosa (Mandhane et al., 2011). When allergens are deposited in the mucous layer of the nasopharynx their water soluble proteins are taken up by these APCs (dendritic cells and macrophages) and processed into short peptides that bind specifically to major histocompatibility complex (MHC) class II molecules (MHCII) expressed on the APCs surface (Bernstein et al., 2016). The APCs migrate to the lymph nodes and present the MHCII peptides to the naïve CD4+ T lymphocytes (Th0). CD4+ lymphocyte activation requires two distinct signals, contact with the MHCII molecules on APCs with specific surface T-cell receptors, and ligation of co-stimulatory receptors CD80 and CD86 on APCs with CD28 family receptors on T cells (Bugeon and Dallman, 2000; KleinJan et al., 2006). Under stimulation with the IL-4 cytokine, activated Th0 lymphocytes are transformed to T helper 2 (Th2) CD4+ cells. Non-atopic subjects can still mount allergen-specific T cell responses to allergen stimulus (Ebner et al., 1995; Van Overtvelt et al., 2008), whereby allergen-specific CD4+ T cells are mainly transformed into IFN-γ producing Th1 cells and IL-10 producing Treg cells (Van Overtvelt et al., 2008). In contrast, T cells in atopic patients are mostly transformed into allergen-specific Th2 cells (Van Overtvelt et al., 2008) which are involved in IgE production. Th2 cells release cytokines IL-4, IL-5 and IL-13 to initiate the inflammatory immune response (Bernstein et al., 2016). Specific B cell subsets are stimulated by IL-4 to differentiate into antibody producing plasma cells. In a process termed ‘isotope switching,’ plasma cells switch production from IgM to IgE antibodies that specifically recognize the allergenic protein. The class switching process is initiated by two signals. The first signal is provided by IL-4 and IL-13 released by T cells (Stone et al., 2010). These cytokines interact with receptors on the B-cell surface and signals induction of ε-germline transcription of B cells to produce IgE antibodies and successive clonal expansion of IgE expressing memory B cells (Sin and Togias, 2011). The second signal is a costimulatory interaction between CD154 (CD40 ligand) on the surface of activated T cells with the CD40 molecule expressed on the surface of B cells (Janeway et al., 2001). This second signal stimulates B cell activation and class switch recombination to induce IgE production (Sin and Togias, 2011).

IgE antibodies represent a very small fraction of the total antibody concentration in human serum (Bernstein et al., 2016). However, on binding with specific cell surface receptors and cross-linking with antigen, IgE can induce powerful inflammatory effects. Allergen specific IgE antibodies bind strongly with high affinity receptors (FcεRI) expressed on the surface of mast cells and basophils (Kraft and Kinet, 2007), which are abundant in the nasal mucosa. On re-exposure to allergen, the specific allergenic protein is recognized by the IgE antibodies bound to FcεRI receptors. On cross-linking of many dimeric or higher order oligomeric receptor molecules (Fewtrell and Metzger, 1980; Knol, 2006), a sequence of reactions is initiated, leading to the degranulation of mast cell and basophil vesicles and release of histamine, platelet activating factor and tryptase (Norman et al., 1985; Bernstein et al., 2016). Activated mast cells also release arachidonic acid from membrane stores, which is a precursor to the eicosanoid synthetic pathway, involved in the production of cysteinyl leukotrienes (LTC_4_, LTD_4_, and LTE_4_) and prostaglandins (primarily PGD_2_) (Peters-Golden et al., 2006).

### Early Phase Response

**FIGURE 1:**
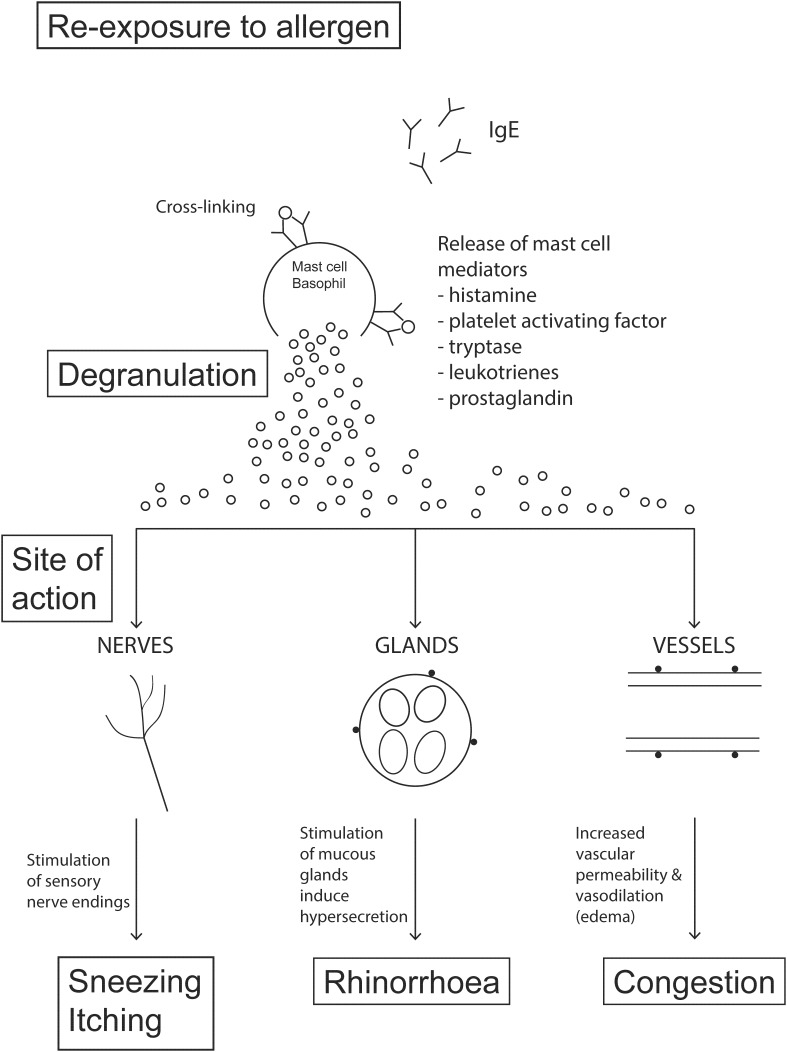
The early phase response. Crosslinking of FCεR1-bound IgE antibodies on the mast cell surface in response to secondary allergen exposure stimulates the degranulation of mast cells. Degranulation induces the release of chemical mediators (primarily histamine) that stimulate sensory nerve endings, mucous glands and small vessels of the nasal mucosa to produce classic rhinitis symptoms: sneezing, nasal itching, rhinorrhoea and nasal congestion. The onset of action is typically within minutes of exposure and is sustained for 2–3 h forming the early-phase response.

Histamine release from mast cells initiates the early or immediate phase response (Figure 1), typically occurs within 1 min of allergen exposure, and can last greater than 1 h (Wang et al., 1997). The nasal mucosa is innervated by a collection of sensory nerve fibers including Aδ and non-myelinated C fibers, sympathetic, and parasympathetic nerves. Histamine release from mast cells promotes activation of H1 receptors on sensory nerves of the afferent trigeminal system (Doyle et al., 1990; Bachert, 2002). These activated (depolarized) sensory nerves transmit signals to the central nervous system causing itching (Schmelz et al., 1997; Andrew and Craig, 2001) and motor reflexes such as sneezing. Histamine release also stimulates mucous glands to secrete watery discharge, via activation of sensory and parasympathetic nerves, which manifests symptomatically as rhinorrhoea (Al Suleimani and Walker, 2007). Nasal congestion is also caused by histamine release. Histamine stimulates H1 and H2 receptors of nasal blood vessels causing increased vascular permeability and vasodilatation leading to engorgement of blood vessels in the nasal mucosa and the sensation of nasal congestion (Secher et al., 1982; Wood-Baker et al., 1996; Togias, 2003). Histamine release regulates the function of tight junctions in the nasal epithelium via coupling of H1 receptors. This interaction increases paracellular permeability (Flynn et al., 2009; Georas and Rezaee, 2014) which allows APCs to more easily penetrate epithelial tight junctions and augment the antigen capture and processing abilities of APCs. The other mediators released by mast cells and basophils also play a role in smooth muscle contraction, mucous secretion and increased vascular permeability.

### Late Phase Response

**FIGURE 2:**
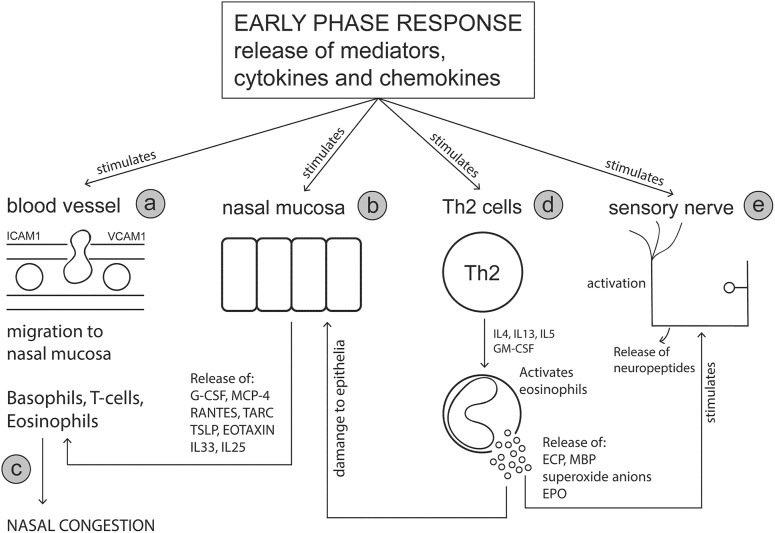
The late phase response. Mediators and cytokines released during the early phase response act on various sites including nasal blood vessels, nasal epithelial cells, T cells and sensory nerves to initiate the symptoms of an allergic response. The late phase response is characterized by the involvement of key immune effector cells including basophils, T cells and eosinophils, which migrate to the nasal mucosa in response to early phase stimulus. The release of cytokines and mediators from these effector cells further perpetuates the allergic response and symptom manifestation. **(a)** Mast cell mediators act on adhesion molecules (e.g., ICAM-1 and VCAM-1) on blood vessel endothelial cells increasing vascular permeability thereby allowing effector cells such as eosinophils, T cells and basophils to migrate to the nasal mucosa. **(b)** Nasal mucosal cells are stimulated by mast cell products to secrete cell signaling molecules which further promote chemoattraction of effector cells to the nasal mucosa. **(c)** Nasal edema (congestion) is worsened by the influx of immune cells and their subsequent mediator release. **(d)** Cytokine release from T cells, activates and stimulates eosinophils to release toxic mediators. **(e)** Eosinophil derived mediators damage the nasal epithelium and leave nerve fibers exposed to histamine and other mediators promoting neurogenic inflammation. G-CSF, Granulocyte-colony stimulating factor; MCP-4, Monocyte chemotactic protein-4; RANTES, Regulated on activation normal T cell expressed and secreted; TARC, Thymus- and activation-regulated chemokine; TSLP, Thymic stromal lymphopoietin, GM-CSF, Granulocyte-macrophage colony-stimulating factor; ECP, Eosinophil cationic protein; MBP, Major basic protein.

The primary effector cells of the early phase response (mast cells and basophils) release cytokines and chemokines which attract additional cell types to the nasal mucosa, including eosinophils, Th2 cells, group 2 innate lymphoid cells (ILC2s) and neutrophils (Sin and Togias, 2011). The late phase response (Figure 2) is characterized by an influx of these migratory immune cells and the subsequent release of additional cytokines and mediators from these cells which sustains inflammation and prolongs symptoms (Mandhane et al., 2011; Pawankar et al., 2011). The late phase reaction typically occurs between 4 and 5 h after initial allergen exposure and can last up to 24 h. Whilst symptoms of rhinorrhoea and sneezing persist, ongoing nasal congestion is typically indicative of a late phase reaction (Bousquet et al., 2001). Nasal biopsy specimens and nasal lavage samples collected during the allergy season, or under experimental stimulations using nasal allergen provocation tests, have shown that immune cells such as basophils, eosinophils, neutrophils, mast cells, CD4+ T cells and macrophages (Bascom et al., 1988a,b; Bentley et al., 1992; Fokkens et al., 1992; Lim et al., 1995; Durham et al., 1996; Godthelp et al., 1996; Pawankar et al., 2011) are increased in the nasal mucosa. It is noted that the presence of these immune cells was found to vary depending on the method of nasal mucosa sampling and the time the samples were taken (i.e., in or out of allergy season and timepoint after initial allergen provocation).

The late phase response is a highly complex pathophysiology involving various cytokines, chemokines and mediators released from different cell types, which interact together to perpetuate the allergic response. Mast cells release cytokines such as IL-4, IL-13 and TNF-α that play a role in activation of endothelial cells and upregulate expression of adhesion molecules such as (ICAM-1, VCAM-1) to allow eosinophils, T cells, basophils and neutrophils to migrate to the nasal mucosa (Okano, 2009; Pawankar et al., 2011; Amin, 2012). Release of mediators from mast cells, such as leukotrienes, prostaglandins and platelet activating factor, are responsible for inducing symptoms as well as possessing chemoattractant abilities (Bernstein et al., 2016). In particular, cysteinyl leukotrienes and prostaglandin D_2_ released from mast cells are responsible for recruitment and activation ILC2 cells (Doherty et al., 2013; Chang et al., 2014). Indeed, elevated numbers of ILC2 been identified in peripheral blood (Doherty et al., 2014; Lao-Araya et al., 2014) and nasal mucosal samples (Dhariwal et al., 2017) from AR subjects during the pollen season or following nasal allergen challenge. Upon activation, ILC2 cells release large amounts of Th2 cytokines within the mucosal tissue which further aids to sustain inflammation (Zhong et al., 2017; Doherty and Broide, 2019).

The role of neutrophils in allergic inflammation is being increasingly recognized (Fransson et al., 2004; Hosoki et al., 2016; Arebro et al., 2017). Neutrophils recruited to the nasal mucosa, produce compounds such as reactive oxygen species, proteases such as elastase, and enzymes including metallopeptidase 9 and myeloperoxidase (MPO) which contribute to epithelial damage and recruitment of effector cells to the nasal mucosa (Monteseirin, 2009). Recent evidence suggests that neutrophils under stimulation with cytokines Granulocyte-macrophage colony-stimulating factor (GM-CSF), IFN-γ and IL-3 convert to functional antigen presenting cells and activate allergen-specific effector CD4+ T cells (Polak et al., 2018). The activated T cells contribute to allergic inflammation via the release of IL-5 which activates and recruits eosinophils to the nasal mucosa (Frew and Kay, 1988).

The influx of activated eosinophils to the nasal mucosa is responsible for increased nasal hyperactivity due to exposure of nerve fibers following damage to the epithelium (Ayars et al., 1989). Epithelial damage results from the toxic effects of superoxide anions, hydrogen peroxide production and the release of granular products such as eosinophil cationic protein (ECP), eosinophil derived neurotoxin and major basic protein released from eosinophils (Mandhane et al., 2011). Eosinophils also release IL-5, which acts in an autocrine manner to promote the activation and survival of eosinophils (Akuthota and Weller, 2012). T cells and mast cells also contribute to survival of eosinophils in the nasal mucosa via release of GM-CSF and IL-5 (Yamaguchi et al., 1991; Park et al., 1998).

Direct allergen exposure as well as mediator and cytokine release from primary effector cells (mast cells, basophils and T cells) can also stimulate structural cells in the nasal mucosa, including fibroblasts and epithelial cells, to release additional inflammatory chemokines and cytokines (Sin and Togias, 2011). Epithelial cells and fibroblasts are stimulated to release cytokines and chemokines such as Regulated upon Activation, Normal T cell Expressed, and Secreted (RANTES), thymus and activation regulated chemokine, thymic stromal lymphopoietin, eotaxin, IL-33, IL-25, granulocyte colony-stimulating factor and monocyte chemoattractant protein 4 (MCP-4). These pro-inflammatory molecules act as chemoattractants to augment the Th2 response and contribute to the recruitment of eosinophils, basophils and T cells to the nasal mucosa (Takahashi et al., 2006; Pawankar et al., 2011; Bernstein et al., 2016).

### Priming Effect

Increased nasal symptoms have been reported in subjects at the end of the pollen season, despite similar levels of aeroallergens (Norman, 1969). This observation is known as the ‘priming effect.’ Priming to allergen refers to the occurrence of increased nasal reactivity to allergens following repeated allergen exposure and has been confirmed under experimental allergen challenge models (Connell, 1969; Wachs et al., 1989). It is believed that priming to allergen occurs in response to chronic allergen exposure, whereby increased numbers of immune cells migrate to the nasal mucosa (particularly basophils) providing additional sites for IgE – allergen interaction and mediator release (Wachs et al., 1989; Bousquet et al., 1996).

### Endotypes of Rhinitis

The assessment of the pathophysiology of allergic disease has changed from a generic focus on symptoms and tissue function, to the recognition of complex immune-regulatory networks that underpin the unique clinical presentation observed between individuals with allergic disease. Rhinitis is classically divided into 3 major clinical *phenotypes*, that is, grouping based on distinct clinical observations, these include: infectious rhinitis, non-infectious, non-allergic rhinitis (NAR) and allergic rhinitis with a combination of phenotypes present in some patients (Papadopoulos et al., 2015). Disease classification based on *endotypes*, that is, based on a distinct pathophysiological mechanism, has been recently proposed and is extensively reviewed elsewhere (Papadopoulos et al., 2015; Agache and Akdis, 2016; Muraro et al., 2016; Agache and Rogozea, 2018). Briefly, the endotypes described for rhinitis include: *Type two inflammation*, associated with the presence of eosinophils/ECP release, IgE and cytokines IL-5, IL-4 and IL-13 and seen in patients with AR, chronic rhinosinusitis and nasal polyposis; *Non-type two inflammation*, associated with neutrophils/MPO release, cytokines INF-γ, TNFα, IL-1P, IL-6 and IL-8 and seen in patients with infectious rhinitis; *Neurogenic endotype*, associated with over expression of transient receptor potential (TRP) channels, nasal hyperactivity and high concentrations of neurokinins and substance P, and is seen in patients with idiopathic rhinitis and gustatory rhinitis; and *Epithelial dysfunction*, associated with reduced expression of tight junction proteins, enhanced subepithelial migration of exogenous antigenic molecules and is seen in patients with AR, infectious rhinitis and chronic rhinosinusitis with or without nasal polyps (Agache and Akdis, 2016; Muraro et al., 2016). It has been proposed that endotype classification may explain the variation observed between patients in clinical presentation and treatment response (Papadopoulos et al., 2015).

## Intranasal Pharmaceutical Treatment of Allergic Rhinitis

The presence of AR symptoms is associated with allergen exposure. Strategies employed to avoid allergen exposure such as staying indoors with closed windows or wearing a mask is highly impractical and is not widely practiced (Kemp, 2009). The rationale for using intranasal application of medications in the treatment of AR, is that high doses of drug can be applied directly toward receptor sites at the source of inflammation (nasal mucosa) with minimal risk of systemic side effects (Bousquet et al., 2008). Many drugs, which act via different mechanisms, have been developed for intranasal application. Antihistamines and corticosteroids are the most commonly used intranasal medications for AR symptoms. Other medications such as decongestants, anticholinergics and chromones have also been formulated for intranasal application, however they are only modestly effective and are recommended as an adjunct therapy or for mild symptoms (Bousquet et al., 2008).

## Intranasal Antihistamines

The interaction of histamine with H1 receptors is the primary cause for manifestation of early phase allergic responses that manifest as rhinorrhoea, itch and contraction of bronchial smooth muscles (Leurs et al., 2002). Antihistamines act on histamine receptors to ameliorate the effects of histamine by stabilizing the receptor in an inactive conformation. Azelastine hydrochloride and olopatadine hydrochloride are the only two intranasal antihistamine (INAH) spray formulations to be approved by the Food and Drug Administration for relief of AR symptoms.

The pharmacological profile and clinical efficacy of azelastine hydrochloride and olopatadine hydrochloride have been extensively reviewed elsewhere (Bernstein, 2007; Horak, 2008; Berger, 2009; Horbal and Bernstein, 2010; Kaliner et al., 2010). Both drugs are classed as second-generation antihistamines with high affinities for the H1 receptor and little affinity for the H2 receptor (Sharif et al., 1996; Bernstein, 2007). Intranasal antihistamines typically have a fast onset of action, demonstrated to significantly reduce symptoms within 15 to 30 min (Horak et al., 2006; Patel et al., 2007a,b) with effects lasting up to 12 h (Greiff et al., 1997; Patel et al., 2007c). INAH are more effective at reducing symptoms of itching, rhinorrhoea and sneezing compared to oral antihistamines, but are less effective at reducing concurrent ocular symptoms (Corren et al., 2005; Bousquet et al., 2008). Like an oral antihistamine, INAH therapy typically has variable effects on nasal congestion (Golden and Craig, 1999; Bousquet et al., 2008).

### Mechanisms/Modulation

**FIGURE 3:**
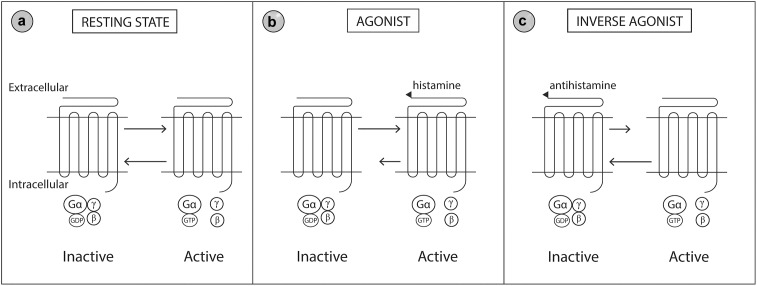
Molecular model of the histamine 1 (H1) receptor. The H1 receptor is a G protein-coupled transmembrane receptor which acts as a ‘molecular switcher’ via interactions with their associated intracellular heterotrimeric G proteins (consisting of α, β, and γ subunits). G proteins regulate downstream intracellular signaling via their ability to catalyze the exchange of Gα bound GDP to GTP. The H1 receptor complex exists between two conformational states, active and inactive, which are directed by specific extracellular ligand binding to the G protein receptor. **(a)** When the active and inactive state are in equilibrium, the H1 receptor is in a resting state. **(b)** Histamine (an agonist) binds to and stabilizes the receptor in the active conformation which shifts the equilibrium toward the active state. **(c)** Antihistamines (an inverse agonist) binds to and stabilizes the receptor in the inactive conformation which shifts the equilibrium toward the inactive state. Gβ, Guanine nucleotide-binding protein beta; Gγ, Guanine nucleotide-binding protein gamma; Gα, Guanine nucleotide-binding protein alpha; GDP, Guanosine diphosphate; GTP, Guanosine triphosphate. Modified from Simons and Simons (2011).

The H1 receptor is widely distributed throughout the body. Expression of the H1 receptor has been documented in smooth muscle, heart, adrenal medulla, sensory nerves, central nervous system, epithelial cells and immune endothelial cells (Mahdy and Webster, 2011). Histamine receptors are heptahelical G-protein coupled transmembrane receptors that transduce extracellular signals through G proteins to intracellular second messenger systems (Simons and Simons, 2011). Histamine receptors may be considered a ‘cellular switcher,’ functioning in equilibrium between two conformation states, active or inactive (Figure 3). Antihistamine drugs are classified as inverse agonists, as they are not structurally related to histamine and do not antagonize the binding of histamine, but instead bind to different sites on the receptor (Wieland et al., 1999; Gillard et al., 2002). Binding of antihistamines to the histamine receptor stabilizes the receptor in the inactive state thereby reducing the intrinsic activity of the receptor in response to histamine (Mahdy and Webster, 2011; Simons and Simons, 2011).

**FIGURE 4:**
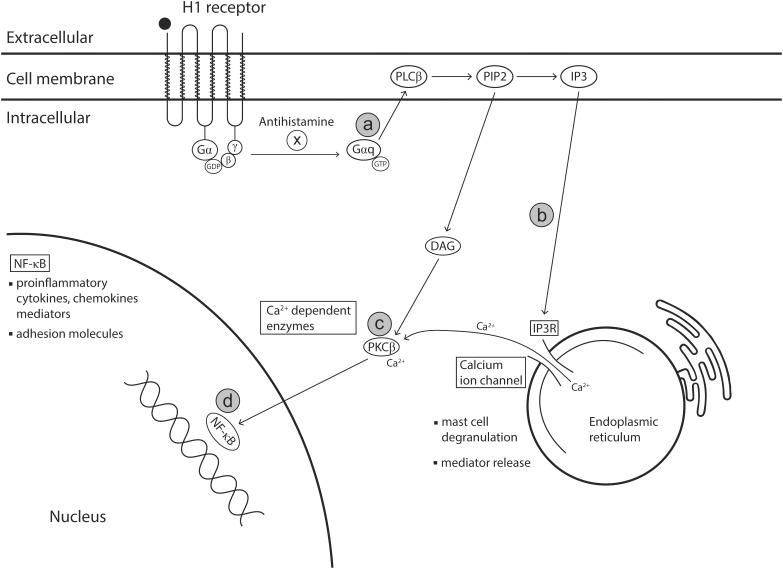
Anti-inflammatory effects of antihistamines. Binding of antihistamines (an inverse agonist) to the transmembrane H1 receptor prevents the activation of intracellular signaling pathways that result in mast cell degranulation and NF-κB activation. Alternatively, when histamine (an agonist) binds to the H1 receptor, this signals the associated G protein subunit Gαq to activate the phospholipase C and phosphatidylinositol (PIP2) signaling pathways. **(a)** Gαq activates phospholipase C which cleaves phosphatidylinositol 4,5-bisphosphate (PIP2), a phospholipid constituent of the cell membrane, into diacyl glycerol (DAG) and inositol 1,4,5 triphosphate (IP3). **(b)** IP3 is then released into the cytoplasm where it binds to IP3 receptors situated in the endoplasmic reticulum (ER). The IP3 receptors are intracellular channels that facilitate calcium ion release. On binding with IP3, IP3 receptors are stimulated to release calcium ions from ER stores into the cytosol. Mast cell degranulation and subsequent mediator release is dependent on this flux in calcium ion availability in the cytosol. **(c)** Calcium ions and DAG (cleaved from PIP2) activate protein kinase C which is involved in activating the transcription factor NF-κB. **(d)** Activation of NF-κB results in increased transcription of proinflammatory genes. DAG, 1,2-diacyl-glycerol; PLCβ, phospholipase C β, PIP2, phosphatidylinositol 4,5-biphosphate; IP3, Inositol 1,4,5-triphosphate; IR, Inositol 1,4,5-triphosphate receptor type 1; PKCβ, protein kinase C beta; NADPH, nicotinamide adenine dinucleotide phosphate. Modified from Frolkis et al. (2010); Simons and Simons (2011), and Jewison et al. (2014).

While histamine is an important mediator involved in the pathophysiology of the allergic response, other mediators released from various immune cells such as leukotrienes, prostaglandins, kinins, cytokines, platelet-activating factor (PAF) and ECP, are responsible for amplifying and maintaining inflammation and therefore prolonging symptoms. There is some evidence to suggest that specific antihistamines including azelastine hydrochloride and olopatadine hydrochloride can exert anti-allergic effects beyond inhibiting the action of histamine on histamine receptors (Figure 4).

### Action on Histamine Receptors

In stimulated cell culture models, azelastine hydrochloride treatment reduced secretion of pro-inflammatory cytokines TNF-α (Hide et al., 1997; Yoneda et al., 1997; Matsuo and Takayama, 1998), IL-1β (Yoneda et al., 1997), GM-CSF (Yoneda et al., 1997) and IL-6 (Yoneda et al., 1997; Kempuraj et al., 2002). Similarly, *in vitro* studies of olopatadine hydrochloride treatment indicate reduced secretion of pro-inflammatory cytokines RANTES (Yamauchi et al., 2007), TNF-α (Cook et al., 2000), IL-6 (Yanni et al., 1999; Kempuraj et al., 2002), IL-8 (Yanni et al., 1999) and chemokine MCP-1 (Yamauchi et al., 2007). Further, there is some evidence to suggest that azelastine hydrochloride and olopatadine hydrochloride may also influence the production of eicosanoids. In a cell culture model using A23187 stimulated rat basophilic leukemia (RBL)-1 cells, Hamasaki et al. (1996) reported that azelastine treatment inhibited leukotriene C_4_ production via inhibition of phospholipase A_2_ and leukotriene C_4_ synthase. The mechanisms behind these reported anti-inflammatory effects have not been fully described. It has been suggested that antihistamines may interfere with the constitutive signaling pathway between the H1 receptor and the ubiquitous transcription factor nuclear factor kappa B (NF-κB) (Leurs et al., 2002; Canonica and Blaiss, 2011), which is involved in the expression of pro-inflammatory cytokines, cell adhesion molecules and chemotaxis of inflammatory cells (Barnes and Karin, 1997; Simons and Simons, 2011). However, it is noted that while these studies reported dose-dependent effects, the concentrations of drugs used may not align with the physiological levels achieved by therapeutic administration.

Many clinical trials have been conducted to assess efficacy of olopatadine hydrochloride (Kaliner et al., 2010), however, few *in vivo* studies have evaluated its mechanism of action. In a sensitized guinea pig model, Kaise et al. (2001) reported reduced thromboxane A_2_ (TXA_2_) concentration in the nasal lavage fluid following oral administration of olopatadine. This result is consistent with the findings of rat cell-culture models exhibiting reduction in Leukotriene C_4_ (Chand et al., 1989; Hamasaki et al., 1996), which is derived from the same arachidonic acid pathway as thromboxane. Saengpanich et al. (2002) did not report any significant reduction in late-phase (24 h post allergen challenge) cytokines including IL-4, IL-5 and TNF-α in nasal lavage fluid following intranasal administration of azelastine hydrochloride (548 μg/day). Interestingly, this contradicts reports of reduced TNF-α production in cell culture models of human monocytes, and mouse and rat mast cells following treatment with azelastine (Hide et al., 1997; Yoneda et al., 1997; Matsuo and Takayama, 1998). Unlike isolated cell culture, nasal lavage fluid contains a variety of cell types including epithelial cells which may exhibit differential TNF-α expression. In addition, the method of application of azelastine drug (i.e., applied directly to the nasal mucosal tissue vs. to isolated immune cells) may influence the ability of azelastine to inhibit TNF-α. These key differences in experimental design, may explain the discordant results between *in vitro* and *in vivo* reports. Additional *in vivo* studies are certainly warranted to clarify these effects observed.

### Alternative Mechanisms – Non-histamine Receptor Mediated

Anti-inflammatory activities independent of the H1 receptor have also been reported for azelastine hydrochloride and olopatadine hydrochloride. The mechanisms behind this action have not been fully elucidated, but may involve interference with calcium ion channels, thereby reducing the intracellular calcium ion accumulation in mast cells needed to elicit degranulation (Letari et al., 1994). In support of this theory, *in vitro* stimulated cell culture models have shown reduced histamine (Norman, 1969; Bernstein, 2007; Kaliner et al., 2010) and tryptase (Norman, 1969) release from mast cells following treatment with azelastine or olopatadine. This disruption of calcium ion channels may also inhibit the production of calcium-dependent enzymes such as protein kinase C (PKC) and NADPH oxidase which are involved in synthesis and release of pro-inflammatory mediators (Umeki, 1992; Leurs et al., 2002; Walsh, 2005; Simons and Simons, 2011).

Clinical studies assessing histamine and tryptase release under allergen challenge following treatment with azelastine hydrochloride or olopatadine hydrochloride yielded inconsistent results. Jacobi et al. (1999) were the first to report positive findings, noting a significant reduction in allergen-associated increases in histamine and tryptase levels in nasal lavage fluid following pre-treatment with azelastine hydrochloride nasal spray (0.14 mg/nostril, twice daily) at prescribed doses for AR treatment. In contrast, Shin et al. (1992) reported no significant reduction in histamine concentration in nasal lavage fluid following a single oral 2 mg dose of azelastine hydrochloride. Similarly, Saengpanich et al. (2002) reported no significant reduction in histamine or tryptase levels in nasal lavage fluid following allergen challenge and pre-treatment with a commercially available azelastine hydrochloride nasal spray (548 μg/day) for 2 weeks at approved dosage. In a subsequent study, Pipkorn et al. (2008) compared the effects of pre-treatment with azelastine (0.1%) and olopatadine (0.1%) nasal sprays on histamine release following allergen challenge in adult AR sufferers. The authors did not report a significant reduction in histamine concentration in nasal lavage fluids following pre-treatment with azelastine (0.1%). A similar effect on histamine release was observed with the same concentration of olopatadine. Interestingly, at a higher concentration of olopatadine (0.2%), a significant reduction in histamine release was reported compared to the placebo. The commercial dosage of olopatadine in a nasal spray formulation is available at 0.6%, which is 3-fold higher than the dosage (0.2%) used in this study. The mixed findings observed across studies may be due to differences in the study design such as allergen challenge duration, nasal lavage collection methods, dose, delivery route and duration of pre-treatment with study drugs. Regardless, these studies were performed in small cohorts of AR sufferers (≤20 subjects) and should be confirmed in larger cohorts.

## Intranasal Steroids

Intranasal corticosteroids are considered the most effective treatment for AR. Corticosteroids suppress many stages of the allergic inflammatory reaction (Bousquet et al., 2001). They have been demonstrated to be more effective for relieving overall AR symptoms than oral and intranasal antihistamines (Weiner et al., 1998; Yáñez and Rodrigo, 2002) and are particularly useful for improving ocular symptoms (DeWester et al., 2003; Anolik et al., 2008) and nasal congestion (Berger et al., 2005).

Systemic corticosteroids, while effective at reducing AR symptoms, pose significant risk of toxicity under long term treatment conditions (Szefler, 2001). In 1972, beclomethasone was the first reported steroid to be effectively modified for use in a pressurized aerosol spray with no apparent systemic drug activity (Brown et al., 1972). Since then, eight compounds for intranasal application have been approved for AR in the United States. These include: triamcinolone acetonide, budesonide, ciclesonide, mometasone furoate, flunisolide, beclomethasone dipropionate, fluticasone propionate and fluticasone furoate (Bousquet et al., 2001; Derendorf and Meltzer, 2008).

### Mechanisms/Modulation

**FIGURE 5:**
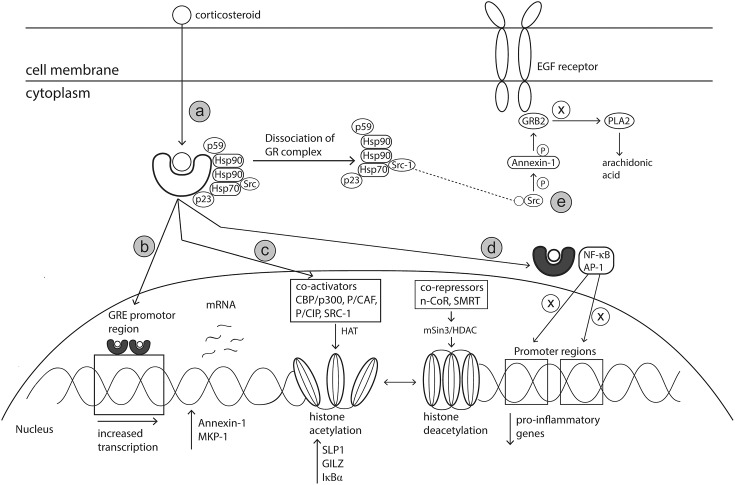
Mechanism of action of corticosteroids. Corticosteroids act via various genomic and non-genomic pathways such as transactivation, transrepression, histone medication and Src kinase signaling, to reduce allergic inflammation. **(a)** Corticosteroids cross cell membranes and bind to a specific intracellular glucocorticoid receptor (GR). The complex of proteins bound to the receptor are released upon receptor-ligand binding, allowing the corticosteroid activated GR to translocate to the nucleus or interact with transcription factors in the cytoplasm. **(b)** Activated GR translocates to the nucleus and binds as a dimer to GRE located within the promotor region of specific anti-inflammatory genes. **(c)** Activated GR can modify chromatin structure to either enhance or prevent transcription of genes via interactions with coactivator and corepressor complexes which have inherent histone acetylation and histone deacetylation abilities, respectively. **(d)** Activated GR can bind directly with transcription factors including AP-1 and NF-κB to prevent binding to their respective promotor regions, thereby preventing the transcription of pro-inflammatory genes. **(e)** SRC, released upon dissociation of GC-GR complex, activates the annexin-1 protein. Annexin-1 then disrupts the signal transduction protein Grb2 which is linked with epidermal growth factor. Impairment of EGF reduces the production of leukotrienes and prostaglandins. Hsp90, heat shock protein 90; GRE, Glucocorticoid response elements; AP-1, activation protein 1; CBP/p300, CREB-binding protein; p/CAF, CBP/p300 associated factor; p/Cip, CBP/p300 co-integrator associated protein; SRC-1, steroid receptor coactivator 1; MKP-1, Mitogen-activated protein kinase 1; SLPI, secretory leukocyte protease inhibitor (SLPI); GILZ, Glucocorticoid-induced leucine zipper; IκBα, nuclear factor of kappa light polypeptide gene enhancer in B-cells inhibitor, alpha; N-CoR, nuclear receptor corepressor; SMRT, silencing mediator of retinoid and thyroid hormone receptor; HDAC, histone deacetylase; PLA2, phospholipase A2.

The primary mode of action of glucocorticosteroids (GC) has been well defined. However, supplementary mechanisms to the primary mode of action have also been postulated. Glucocorticoids readily diffuse across cell membranes where they bind to the cytoplasmic glucocorticoid receptor (GR) (primary mechanism) (Bousquet et al., 2001; Barnes, 2006). The GR, in an inactive state, is comprised of a cytosolic protein bound by a complex of chaperon proteins including heat shock protein (hsp) 90, hsp70 chaperonin molecules, the p59 immunophilin and the small p23 phosphoprotein (Derendorf and Meltzer, 2008). On binding of the GR with the corticosteroid ligand, the heat shock proteins dissociate, allowing the GC-GR complex to translocate into the nucleus or interact with transcription factors in the cytoplasm (Okano, 2009; Figure 5a). The anti-inflammatory effects induced by corticosteroids are the result of modifications to gene transcription occurring via mechanisms known as transactivation or transrepression.

### Transactivation

In the transactivation pathway, the activated GC-GR complex migrates to the nucleus where it binds as a dimer to the promotor region of palindromic DNA sequences termed Glucocorticoid Response Elements (GRE) (Barnes, 2006). Interaction between the activated GR complex and GRE promotes an increase in the transcription of anti-inflammatory genes and of genes encoding proteins that have inhibitory effects on transcription of inflammatory and immune genes (Uva et al., 2012; Figure 5b). GR-GRE binding results in increased expression of Annexin-1, a known inhibitor of phospholipase A_2_. This inhibition of phospholipase A_2_ prevents the synthesis of arachidonate-derived eicosanoids such as thromboxanes, prostaglandins and leukotrienes, which are responsible for perpetuating and worsening AR symptoms. Activation protein-1 (AP-1) is an important transcription factor complex responsible for the transcription of many pro-inflammatory genes such as TNF-α, IL-1, IL-2, IFN-γ, GM-CSF (Trop-Steinberg and Azar, 2017). GR-GRE binding renders the transcription factor AP-1 inactive. This action occurs via up-regulation of MAPK phosphatase 1 (MKP-1) resulting in down-regulation of c-Jun which is an integral component of AP-1 (Barnes, 2006).

### Transrepression

The main anti-inflammatory effects of GCs occur via the suppression of multiple genes that encode inflammatory proteins, a process known as transrepression (Coutinho and Chapman, 2011). Activated GRs modulate gene expression via non-genomic mechanisms such as protein – protein interactions with transcription factors and co-activators rather than binding to the promotor region directly (Coutinho and Chapman, 2011; Figure 5d). Support for the theory of transrepression was based on *in vivo* experiments with dimerization (dim) mutant mice (GR^dim^) (Reichardt et al., 1998, 2001) The glucocorticoid receptor in these GR^dim^ mice did not have ability to dimerise and therefore bind DNA, forcing the GCs to act via alternative non-genomic pathways. After stimulation with LPS and subsequent treatment with steroid dexamethasone, a reduction in inflammatory mediators such as TNF-α, COX-2, IL-6 and IL-1β was observed in both knockout and wildtype mice (Reichardt et al., 1998, 2001). Activated GR proteins interact directly with proteins of transcription factors such as AP-1 and NF-κB resulting in mutual repression where both GR and the transcription factors cannot bind to their relevant DNA promotor regions (Jonat et al., 1990; Ray and Prefontaine, 1994; Tuckermann et al., 1999; Uva et al., 2012). These transcription factors regulate expression of pro-inflammatory cytokines, chemokines (eotaxin, RANTES and MCP), growth factors, adhesion molecules (ICAM-1 and E-selectin), nitric oxide and inflammatory enzymes (Nelson and Ballow, 2003; Uva et al., 2012). Therefore, inactivation of these transcription factors leads to reduced immune cell infiltration and dampening of allergic responses.

### Modifications to Histone Acetylation

Regulation of histone acetylation is another mechanism via which corticosteroids can influence gene expression of a range of key targets relevant in AR pathophysiology. The packaging of DNA into nucleosomes, containing four core histone proteins (H1–H4), prevents the accessibility of transcription factors and therefore the initiation of gene transcription. However, increased histone acetylation results in changes to the nucleosome structure and is associated with increased gene transcription and conversely, hypoacetylation is linked with inhibition of gene transcription (Ura et al., 1997). Glucocorticoids can modify gene transcription through interactions with coactivator proteins or co-repressor complexes which possess histone acetylation and deacetylation activities, respectively (Figure 5c).

Activated GRs increase gene transcription via interactions with coactivator proteins of transcription factors such as cAMP response element-binding protein (CBP/p300), CBP/p300 associated factor (p/CAF), CBP/p300 co-integrator associated protein (p/Cip) and steroid receptor coactivator (SRC-1) (Pelaia et al., 2003; Barnes, 2006). These coactivator proteins have inherent histone acetyltransferase activity which results in the acetylation of core histones H3 and H4, thereby reducing their charge (Barnes and Adcock, 2003; Pelaia et al., 2003). Co-activator proteins also interact with the thyroid hormone receptor associated protein (TRAP) – Glucocorticoid Receptor Interacting Protein (GRIP) – Activated Recruited Cofactor (ARC) complex, designed to recruit core transcriptional machinery (Pelaia et al., 2003). Acetylation of histones changes the nucleosome structure from the resting closed formation to an open formation which allows the transcriptional machinery including RNA polymerase II to bind to DNA and initiate transcription (Barnes and Adcock, 2003; Figure 5c). The histone acetylation process increases the transcription of anti-inflammatory proteins including secretory leukocyte protease inhibitor (SLPI), Glucocorticoid-induced leucine zipper (GILZ) and IκB-α (NF-κB inhibitor) (Barnes, 2006), all of which contribute to ameliorating allergy related inflammation. Indeed, Abbinante-Nissen et al. (1995) demonstrated that SLPI transcript levels were increased in a clear concentration dependent manner in human epithelial cells treated with corticosteroids. Despite these proposed anti-inflammatory effects, this mechanism of action does not occur in all cell types and only in high doses that may not be achieved through therapeutic intervention (Barnes, 2006). As such, alternative mechanisms are likely responsible for in the anti-allergic effects/symptom improvement of corticosteroid treatment.

Gene repression can also occur via the reversal of histone acetylation, a process which is controlled by co-repressor complexes and histone deacetylase enzymes (Adcock, 2003; Figure 5c). Activated GR interacts with co-repressor complexes such as nuclear receptor corepressor (N-CoR) and silencing mediator of retinoid and thyroid hormone receptor (SMRT) (Pelaia et al., 2003; Uhlenhaut et al., 2013). These coactivators recruit the mSIN3-HDAC complex which possess histone deacetylase capabilities (Ito et al., 2000; Pelaia et al., 2003). NF-κB and AP-1 transcription factors are activated during the allergic response and are involved in the transcription of many pro-inflammatory genes. Deacetylation of core histones selectively attenuates the activity of NF-κB and AP-1, via tightening of the chromatin structure and reducing access of these transcription factors to their binding sites, thereby preventing the transcription of pro-inflammatory genes (Ito et al., 2006; Barnes, 2011).

### Alternative Mechanisms

Glucocorticoids can also initiate additional anti-inflammatory effects via alternative mechanisms. In an inactive state, Src kinase binds to GR as part of a protein complex. Once GR is activated by glucocorticoid binding, Src kinase is then released and phosphorylates Annexin-1. Sequentially, Annexin -1 displaces the adaptor protein Grb2 from epidermal growth factor receptor, thereby reducing its activity and inhibiting the activation of cytoplasmic phospholipase A_2_ and its by-product arachidonic acid (Croxtall et al., 2000; Figure 5e). In theory, as arachidonic acid is a precursor for leukotrienes and prostaglandins, this activity should reduce the production of these mediators.

By means of an additional non-genomic mechanism, corticosteroids may reduce the stability of mRNA, thereby inhibiting protein synthesis. Some inflammatory genes such as cyclooxygenase-2 (COX-2) and GM-CSF are particularly susceptible to ribonuclease break down of RNA. Corticosteroids have inhibitory activity toward proteins that stabilize mRNA. Specifically, corticosteroids induce the expression of dual specificity phosphatase 1 (DUSP1), a known inactivator of p38 mitogen-activated protein kinase (MAPK) signaling pathways (Abraham et al., 2006). MAPKs contribute to the expression of proinflammatory genes, including COX-2 (Lasa et al., 2001). Inhibition of MAPK signaling pathways, leaves mRNA of pro-inflammatory genes vulnerable to rapid breakdown and therefore synthesis of these genes is reduced (Barnes, 2006). The transrepression and transactivation mode of action of corticosteroids contributes the most toward improving AR symptoms. Whilst additional mechanisms of corticosteroids on Src kinase and DUSP1 targets have been proposed, it is not known to what degree these mechanisms contribute to symptom relief in AR.

### Experimental Overview of Mechanisms

The anti-inflammatory activity of intranasal steroids has been shown by its effects on several inflammatory mediators and markers both *in vitro* and *in vivo*.

### *In vitro* Studies

Intranasal steroids have been shown to inhibit cytokine production in a range of different cell types. Epithelial generated cytokines act as chemoattractants and recruit effector cells such as eosinophils, basophils and T cells to the nasal mucosa. Treatment with fluticasone propionate or fluticasone furoate significantly reduced levels of GM-CSF, IL-6 and IL-8 in stimulated nasal epithelial cells (Ohnishi et al., 1994; Smith et al., 2002; Mullol et al., 2014). In stimulated murine mast cells, fluticasone propionate was shown to inhibit the release of IL-4, IL-6, IL-8 and TNF-α at an IC_50_ of <1 nM (Fuller et al., 1995). Fluticasone propionate was also shown to significantly reduce IL-4 and IL-5 levels from stimulated peripheral blood CD4+ T cells while a lesser effect was observed on the Th1 cytokine IFN-γ (Umland et al., 1997). It is important to note that there are differences in the degree of cytokine inhibition between classes of steroid drugs. Barton et al. (1991) compared the effect of five steroid drugs on inhibition of IL-6 and TNF-α in LPS stimulated murine myelomoncytic leukemia cells, and inhibition of IL-1 in LPS-stimulated macrophages harvested from the peritoneal cavity of BALB/c mice. Overall, variation in the degree of cytokine inhibition was observed between drugs (mometasone furoate, hydrocortisone, betamethasone, dexamethasone and beclomethasone) with mometasone furoate the most potent inhibitor of IL-1, IL-6, and TNF-α production. Collectively these findings suggest that corticosteroids selectively downregulate Th2 cytokines, rather than Th1 cytokines. As AR is often characterized as a Th2 mediated disease, it is therefore not surprising that corticosteroids are effective treatments for AR.

*In vitro* evidence also suggests that some corticosteroids are effective at inhibiting the maturation, viability and release of mediators from effector cells pertinent to AR. Mast cells are the predominant effector cell involved in the pathogenesis of the early phase response via the release of cytokines and inflammatory mediators such as histamine. In chronic inflammatory conditions, such as the allergic response, mast cells differentiate from bone marrow progenitors, migrate to the site of inflammation and then proliferate and complete maturation in the tissues. Mast cells cultured from human umbilical cord blood mononuclear cells treated with the corticosteroid dexamethasone dose-dependently inhibited the maturation of mast cell progenitors (Smith et al., 2002). Corticosteroids may inhibit the maturation of mast cells via regulating the expression of anti- or pro-apoptotic molecules in mast cell progenitors. In the same experiment, FcεRI dependent release of histamine and cysteinyl leukotrienes from mast cells was unaffected by dexamethasone (Smith et al., 2002). These results indicate that dexamethasone does not modulate the expression of enzymes involved in the synthesis of histamine and cysteinyl leukotrienes in mature mast cells. In contrast, inhibition of histamine release and sulfidoleukotriene production in anti-IgE stimulated basophils was observed following glucocorticoid treatment (Crocker et al., 1997; Stellato et al., 1999). These opposing findings highlight the heterogeneity of corticosteroids in their ability to inhibit histamine and eicosanoid-derived mediator release from different cell types. Eosinophils are key effector cells involved primarily in the late-phase response and are responsible for damage of the airway mucosa and perpetuating the allergic response. The onset of apoptosis of eosinophils can be delayed by inflammatory mediators. Glucocorticoid treatment of eosinophils isolated from whole blood inhibited IL-5 induced eosinophil viability, thereby facilitating apoptosis of eosinophils (Meagher et al., 1996; Stellato et al., 1999). Collectively, the inhibition of mast cell development and eosinophil viability by corticosteroids would likely lead to fewer numbers of mature mast cells and eosinophils in the nasal mucosa, which given the large role these cells play in the allergic reaction, would be expected to aid in symptom resolution should these results be translated to humans.

### *In vivo* Studies

In general, intranasal application of steroids has been found to reduce the numbers of immune cells, production of Th2 cytokines and chemokines and the release of inflammatory mediators in nasal mucosal samples (Table 1). These anti-allergic effects were evident from 1 week of administration. In keeping with *in vitro* observations, corticosteroids seem to actively target Th2 related cytokines (GM-CSF, IL-6, IL-4, IL-5, IL-10 and IL-13) involved in perpetuating the allergic response, in contrast to Th1 cytokines (IFN-γ, IL-2) where no effect of steroid treatment was observed (Table 1).

**Table 1:** Summary of *in vivo* studies examining corticosteroid action on effector cells, cytokines, chemokines and mediators.

CS action	Drug	Dose	Duration	Sample type
**Immune cells**
↓ Eosinophils	Mometasone furoate	200 μg/daily	2 weeks	NL (Ciprandi et al., 2001)
	Fluticasone propionate	100 μg/twice daily	1 week	NL, FP (Erin et al., 2005)
	Fluticasone propionate	100 mg/twice daily)	52 weeks	B (Holm et al., 1999)
	Flunisolide	50 μg/twice daily	1 week	NL (Bascom et al., 1988b)
	Budesonide	100 μg/twice daily	1 week	NL (Benson et al., 2000)
	Fluticasone propionate	200 μg/twice daily	6 weeks	NB, B (Jacobson et al., 1999)
	Fluticasone propionate	200 μg/twice daily	4 weeks	B (Holm et al., 2001)
↓ Activated eosinophils	Fluticasone propionate	200 μg/daily	2 weeks	NL, B (Lozewicz et al., 1992)
↓Neutrophils	Mometasone furoate	200 μg/daily	2 weeks	NL (Ciprandi et al., 2001)
	Flunisolide	50 μg/twice daily	1 week	NL (Bascom et al., 1988b)
↔ Neutrophils	Budesonide	100 μg/twice daily	1 week	NL (Benson et al., 2000)
↓ Basophils	Flunisolide	50 μg/twice daily	1 week	NL (Bascom et al., 1988b)
↓ Langerhans cells	Fluticasone propionate	100 mg/twice daily	52 weeks	B (Holm et al., 1999)
	Fluticasone propionate	200 μg/twice daily	4 weeks	B (Holm et al., 2001)
↓ Mast cells	Fluticasone propionate	100 mg/twice daily	52 weeks	B (Holm et al., 1999)
	Fluticasone propionate	200 μg/twice daily	4 weeks	B (Holm et al., 2001)
↓ T cells	Fluticasone propionate	100 mg/twice daily	52 weeks	B (Holm et al., 1999)
	Fluticasone propionate	200 μg/twice daily	4 weeks	B (Holm et al., 2001)
**Cytokines**
↓ TNF-α	Mometasone furoate	200 μg/daily	2 weeks	NL (Ciprandi et al., 2001)
↔TNF-α	Fluticasone propionate	100 μg/twice daily	1 week	NL, FP (Erin et al., 2005)
	Budesonide	50 μg/twice daily	1 week	NL (Erin et al., 2005)
↓ IL-1α	Fluticasone propionate	100 μg/twice daily	1 week	NL, FP (Erin et al., 2005)
↔ IL-1β	Budesonide	100 μg/twice daily	1 week	NL (Erin et al., 2005)
↓ IL-6	Fluticasone propionate	100 μg/twice daily	1 week	NL, FP (Erin et al., 2005)
	Budesonide	100 μg/twice daily	1 week	NL (Erin et al., 2005)
				
↓IL-13	Fluticasone propionate	100 μg/twice daily	1 week	NL, FP (Erin et al., 2005)
↓ IL-4	Budesonide	100 μg/twice daily	1 week	NL (Erin et al., 2005)
↓ IL-4 expression	Fluticasone propionate	200 μg/twice daily	6 weeks	B (Masuyama et al., 1994)
↓ IL-10	Budesonide	100 μg/twice daily	1 week	NL (Erin et al., 2005)
↓ IL-8	Fluticasone propionate	100 μg/twice daily	1 week	NL, FP (Erin et al., 2005)
				
↓ IL-5	Fluticasone propionate	100 μg/twice daily	1 week	NL, FP (Erin et al., 2005)
↔ IL-5	Mometasone furoate	200 μg, daily	2 weeks	NL (Frieri et al., 1998)
↔ IL-5 expression	Fluticasone propionate	200 μg/twice daily	6 weeks	B (Masuyama et al., 1994)
↔ IL-2	Fluticasone propionate	100 μg/twice daily	1 week	NL, FP (Erin et al., 2005)
↔ IL-3	Fluticasone propionate	100 μg/twice daily	1 week	NL, FP (Erin et al., 2005)
↔ IL-12 (p40)	Fluticasone propionate	100 μg/twice daily	1 week	NL, FP (Erin et al., 2005)
↔IFN-γ	Fluticasone propionate	100 μg/twice daily	1 week	NL, FP (Erin et al., 2005)
	Budesonide	100 μg/twice daily	1 week	NL (Erin et al., 2005)
↔ GM-CSF	Fluticasone propionate	100 μg/twice daily	1 week	NL, FP (Erin et al., 2005)
**Chemokines**
↓ RANTES	Fluticasone propionate	100 μg/twice daily	1 week	NL, FP (Erin et al., 2005)
↓ MCP-1	Fluticasone propionate	100 μg/twice daily	1 week	NL, FP (Erin et al., 2005)
↓ MIP-1α	Fluticasone propionate	100 μg/twice daily	1 week	NL, FP (Erin et al., 2005)
				
↓ IP-10	Fluticasone propionate	100 μg/twice daily	1 week	NL, FP (Erin et al., 2005)
**Mediators**
↓ ECP	Mometasone furoate	200 μg/daily	2 weeks	NL (Ciprandi et al., 2001)
	Fluticasone propionate	200 μg/daily	2 weeks	NL (Lozewicz et al., 1992)
	Budesonide	100 μg/twice daily	1 week	NL (Erin et al., 2005)
↓ Histamine	Mometasone furoate	200 μg, daily	2 weeks	NL (Frieri et al., 1998)
**Adhesion molecules**
↓ Intracellular adhesion molecule -1	Mometasone furoate	200 μg/daily	2 weeks	NL (Ciprandi et al., 2001)
**IgE**
↓ Total IgE	Budesonide	100 μg/twice daily	1 week	NL (Erin et al., 2005)
↔ Total IgE	Beclomethasone dipropionate	400 μg/daily	5 weeks	Blood (Pullerits et al., 1997)
↔ Specific IgE	Beclomethasone dipropionate	400 μg/daily	5 weeks	Blood (Pullerits et al., 1997)


*↑, increased production; ↓, decreased production; ↔, no change; NL, Nasal Lavage; FP, Filter Paper/nasal secretions; B, Nasal Biopsy; NB, Nasal Brushing; References are bracketed.*

## Intranasal Decongestants

The active agents of intranasal decongestants are usually catecholamines (e.g., phenylephrine) or imidazolines (e.g., oxymetazoline) and are classed as vasoconstrictor sympathomimetic agents (Greiner and Meltzer, 2011). These agents exert their decongestion effects through direct and indirect activation of postsynaptic α1- and α2 adrenergic receptors on smooth muscles lining nasal capacitance vessels (Greiner and Meltzer, 2011; Kushnir, 2011; Klimek et al., 2016). On activation of these receptors, the muscles contract, constricting blood vessels and allowing less fluid to leak into nasal tissues (edema) and thus relieving the sensation of nasal congestion (Kushnir, 2011). Intranasal decongestants are effective at rapidly reducing nasal congestion and improving nasal patency (Greiner and Meltzer, 2011; Kushnir, 2011) but have no effect on other symptoms of AR such as nasal itching, rhinorrhoea and sneezing (Bousquet et al., 2008; Gentile et al., 2015; Klimek et al., 2016). With prolonged or repeated use of decongestants (3–5 days) patients may become tolerant to these agents and experience rebound swelling and congestion (Graf, 1999; Gentile et al., 2015).

## Intranasal Anticholinergics

Ipratropium bromide is the only intranasal anticholinergic agent to be commercially available in several countries, including the United States, United Kingdom and Australia (Van Cauwenberge et al., 2000; Bousquet et al., 2008; Tran et al., 2011). Its mechanism of action in the reduction of rhinorrhoea is well recognized (Kaiser et al., 1998). In AR, exposure to allergens stimulates parasympathetic pathways in the nose to release acetylcholine (Kaiser et al., 1998) which acts on muscarinic receptors on nasal mucus glands to induce hypersecretion (Kaiser et al., 1998; Quraishi et al., 2004; Ridolo et al., 2014). Ipratropium bromide is a cholinergic receptor antagonist that blocks the interaction of acetylcholine on muscarinic receptors to inhibit release of watery secretions from mucous glands (Kaiser et al., 1998; Quraishi et al., 2004). Double-blind placebo-controlled studies with AR cohorts have found that ipratropium bromide is effective at reducing severity and duration of rhinorrhoea, but has no effect on symptoms of sneezing or nasal congestion (Borum et al., 1979; Kaiser et al., 1998; Meltzer et al., 2000). Consistent with their singular mechanism of action, no significant changes in proportion of eosinophils, basophils and neutrophils were observed in nasal scrapings following intranasal treatment with ipratropium bromide (21 μg or 42 μg) for 4 weeks (Meltzer et al., 2000).

## Intranasal Chromones

Both cromoglicic acid, a derivative of chromone-2-carboxylic acid and nedocromil sodium, a pyranoquinolone, are available as intranasal formulations. Chromones are considered effective in relieving symptoms of nasal itching, rhinorrhoea and sneezing. However, they have no effect on nasal congestion (Gentile et al., 2015). Their duration of action is short, requiring frequent dosing (up to four times per day) (Barnes, 2000; Gentile et al., 2015).

The exact mechanism of action of chromones is unknown, although several theories have been postulated. Chromones are thought to exert their anti-inflammatory effects by preventing the release of histamine, tryptase and leukotrienes from mast cells following binding of IgE antibodies to the FcεRI receptor and crosslinking with allergenic peptides (Leung et al., 1988; Shichijo et al., 1998; Ridolo et al., 2014). Chromones also have reported effects on other effector cells involved in the allergic response. Nedocromil sodium at 10^-5^ mol/L inhibited the release of ECP, peroxidase and arylsulphatase from cultured eosinophils (Spry et al., 1986) and at the same concentration inhibited lysozyme secretion from rat peritoneal neutrophils (Bradford and Rubin, 1986). In an *in vivo* study, intranasal application of sodium cromoglicic acid (4%) four times daily for 4 weeks, significantly decreased eosinophil counts, but had no significant effect on basophils or neutrophils (Orgel et al., 1991).

There is increasing evidence to suggest that chromones may act on certain types of chloride channels expressed in immune cells which may explain their cell membrane stabilizing effects. Degranulation of mast cells requires the sustained elevation of intracellular calcium stores. Cromoglicic acid and nedocromil sodium have been shown to inhibit calcium channel activation following antigen crosslinking with IgE bound to cell membranes (Bousquet et al., 2008; Van Overtvelt et al., 2008; Stone et al., 2010).

Other alternative mechanisms of action beyond chloride channel disruption have been postulated for chromones and include targeting the Annexin-A1 system and activation of the G Protein Coupled Receptor 35 (GPR35). Like glucocorticoids, experimental animal models, have provided evidence that chromones act on the annexin-A1 pathway to achieve therapeutic benefit (Bandeira-Melo et al., 2005; Yazid et al., 2013; Sinniah et al., 2016). Annexin-A1 suppresses phospholipase A_2_ activity and thereby prevents eicosanoid production (leukotrienes and prostaglandins) which are major mediators of the inflammatory response. While nedocromil treatment (10 nM) inhibited the release of mediators in murine bone marrow derived mast cells stimulated with compound 48/80 or IgE/anti-IgE, this effect was not observed in Anx-A1 null mice or in the presence of anti-Anx-A1 antibodies (Yazid et al., 2013). Chromones promote the phosphorylation, externalization and release of annexin-A1 from the cell via PKC activation (Yazid et al., 2013; Sinniah et al., 2017). On release, annexin-A1 can then bind and activate receptors of the Formyl Peptide Receptor (FPR) family (Walther et al., 2000; Sinniah et al., 2016) situated on mast cells. This binding action then inhibits the degranulation of mast cell vesicles in response to stimuli. It has been postulated that the regulation of PKC activity is restricted by Protein Phosphate 2A (PP2A). Chromones are thought to be inhibitors of PP2A. PP2A prolongs the action of PKC and as a result further promotes Annexin-A1 release (Yazid et al., 2013; Sinniah et al., 2016).

While the action of Chromones via the annexin A1 system is rapid (occuring within 5 min), the GPR35 activation pathway takes longer to illicit any therapeutic effect (Sinniah et al., 2017). GPR35 is a G-coupled protein receptor and is present in human mast cells, eosinophils and basophils and modulates signaling via the Gi pathway (Jenkins et al., 2010; Yang et al., 2010; Yazid et al., 2013). While it has been long suggested that products of tryptophan metabolism, such as kynurenic acid (Yang et al., 2010), are ligands for this receptor, other potential roles for this receptor have been hypothesized in recent years (Divorty et al., 2015). Two main studies have reported that chromones are potent GPR35 agonists (Jenkins et al., 2010; Yang et al., 2010). However, it is not known what effect this interaction has on mediator release or its relevance to allergic disease (Yazid et al., 2013; Sinniah et al., 2017).

## Combination Therapy

Survey results published in 2012 showed that 70.5% of moderate/severe AR sufferers in the United Kingdom require multiple therapies to achieve effective symptom relief during the pollen season (Pitman et al., 2012). In addition, many physicians reportedly prescribe multiple therapies to achieve more comprehensive symptom relief (Demoly et al., 2002; Canonica et al., 2007; Schatz, 2007). On this basis, combination nasal sprays have been developed to meet demands for better control of symptoms in the convenient and cost-effective form of a single spray. Combination nasal sprays containing antihistamines and corticosteroids have been the most extensively studied and commercialized. Only one study examining combination nasal sprays containing an anticholinergic and a steroid has been conducted.

### Intranasal Steroids and Antihistamines

In randomized placebo-controlled studies of AR cohorts, head-to-head comparisons of each active ingredient versus the combination (azelastine hydrochloride vs. fluticasone propionate and olopatadine hydrochloride vs. mometasone furoate) revealed that in all studies, the combination was more effective than either monotherapy based on symptom scores (Ratner et al., 2008, 2017; Hampel et al., 2010; Meltzer et al., 2012; Fokkens et al., 2015). To this effect, the data generated from three multi-center head-to-head (fluticasone propionate vs. azelastine hydrochloride) comparison trials of in cohorts of moderate-to-severe seasonal AR sufferers was compiled in a meta-analysis. Average change from baseline total nasal symptom score (severity of rhinorrhoea, sneezing, nasal itching and congestion) was significantly greatest in the combination group (-5.7 ± 5.3, mean ± SD) followed by fluticasone propionate (-5.1 ± 4.9) and azelastine hydrochloride (-4.4 ± 4.8) (Carr et al., 2012). Interestingly, in a recent systematic review the combination of an intranasal antihistamine and corticosteroid was found to be more effective than monotherapy with the corticosteroid, these effects however were not observed with the combination of an oral antihistamine and intranasal steroid (Seresirikachorn et al., 2018). The authors of this paper suggest that intranasal application allows for higher doses of antihistamine drugs to be applied to the site of inflammation which could provide greater antihistaminic action and symptom improvement.

Given that antihistamines and corticosteroids have a separate mechanism of action, it is possible that the enhanced effects observed when combined, may be due to additive or synergistic actions. Few experimental studies have examined the mechanisms of action of the combination effect, despite the reported superior alleviation of symptoms. Mechanistic studies to date have examined the effect of the combination treatment on adhesion molecules and T cell subsets. Increased expression of ICAM-1 is associated with enhanced migration of inflammatory cells (Wegner et al., 1990) into the nasal mucosa. The combination of azelastine hydrochloride and budesonide synergistically increased MKP-1 mediated ICAM-1 inhibition in stimulated cultured nasal epithelial cells, compared to either monotherapy (Luo et al., 2015). These findings were confirmed in a small subset of AR subjects whereby 2-week administration of the combination spray was found to significantly inhibit ICAM-1 expression in nasal mucosal samples when compared to budesonide alone (Luo et al., 2015). In an *in vivo* murine AR model Kim et al. (2017) used *Dermatophagoides farinae* sensitized BALB/c mice to examine the effect of antihistamine treatment (azelastine hydrochloride), steroid treatment (mometasone) or combination treatment on the expression of specific T cell subset markers. Following allergen challenge, the combination therapy was reportedly more effective at reducing ROR-γt (Th17) expression in the murine mucosa compared to mometasone alone, however no superior effect over azelastine treatment alone was observed. In addition, the combination treatment was not significantly more effective than monotherapy at improving Th1/Th2 balance, quantified via expression of IFN-γ and T-bet (Th1) and GATA3 and IL-4 (Th2) cell-specific markers (Kim et al., 2017).

Both steroids and antihistamines reportedly interfere with the ubiquitous transcription factor NF-κB (Leurs et al., 2002; Canonica and Blaiss, 2011; Uva et al., 2012) thereby preventing the expression of pro-inflammatory genes that contribute to AR symptom manifestation. However, it is not known whether the combined therapy would further enhance inhibition of NF-κB activity. Given the multiple mechanism of action of between antihistamines and steroids, future studies should employ a broad analytical approach in identifying potential synergistic targets of the combination therapy.

### Other Combination Sprays

In a double-blind, placebo controlled study in a cohort of AR and non-AR participants, 2 week intranasal administration of the ipratropium bromide (42 μg per nostril, three times daily) plus beclomethasone dipropionate (84 μg per nostril, twice daily) was more effective than either monotherapy alone improving control of rhinorrhoea (73% combination vs. 65% ipratropium bromide monotherapy vs. 68% beclomethasone dipropionate; proportion of participants reporting good or excellent control of rhinorrhoea).

While, to our knowlegde, a commerical chromone and steroid nasal spray is not currently available, synergistic effects between steroids and chromones have been noted. Corticosteroids increase PKC activation and subsequent release of intra-cellular annexin A1. In a concentration dependent manner, annexin A1 release was greatly enhanced with the combination of both chromones and corticosteroids, resulting in greater inhibition of thromboxane (Tx) B_2_ generation (Yazid et al., 2009).

## The Future of Pharmacotherapy in Allergic Rhinitis

The prospect for intra-nasal drugs to treat AR symptoms in the future may include additional combination treatments. Combination therapies should, in theory, cover a broader range of inflammatory pathways and symptoms. Significant symptom improvement was achieved with a steroid (fluticasone propionate) and antihistamine (azelastine hydrocholoride) combination spray (Carr et al., 2012). These positive findings may spur additional mixes of steroid and antihistamine compounds (e.g., olopatadine and budesonide) to be commercially developed into a single product. The combination of an oral monteluskast and an intranasal steroid was shown to be more effective at improving nasal congestion compared with either treatment alone (Chen et al., 2018). An intranasal formulation of montelukast sodium is currently being investigated (Jullaphant et al., 2018) and may be marketed as a stand alone treatment or incorporated as a combination spray with either anthistamines or steroids. There is also the potential for a chromone and steroid combination spray to be developed. The observed improvements in ocular and nasal symptoms and comparable safety profile of the double-dose steroid compared to the single dose (Khattiyawittayakun et al., 2018), may support the development and use of higher dose steroid sprays and inform changes to the recommended treatment practice.

Novel nasal sprays targeting different components of the allergic response, such as histamine 3 and 4 receptors and local sensory nerves are currently in development. The role of other histamine receptors (H3 and H4) in the allergic response is being increasingly recognized, and drugs that target these receptors are currently in clinical development. H3 histamine receptors are present in the brain and nasal mucosa (Barchuk et al., 2013). H3 receptor antagonists are thought to act as nasal vasoconstrictors thereby reducing nasal congestion (Stokes et al., 2012). While oral H3 agonists trialed in humans to date have shown improved efficacy for symptom improvement over placebo (Daley-Yates et al., 2012; North et al., 2014), they have not yet been shown to be as effective at reducing symptoms than currently available therapies pseudoephedrine (decongestant) or H1 antihistamines (Daley-Yates et al., 2012; Barchuk et al., 2013; North et al., 2014). H4 histamine receptors are widely expressed in cells and tissues of the immune system and the also the CNS. H4 receptors have been associated with dendritic cell activation, T cell differentiation and chemotaxis of eosinophils and mast cells. H4 receptor antagonists have shown promising anti-inflammatory and anti- pruritus effects in cell-culture and in animal models (Lazewska and Kiec-Kononowicz, 2012). In addition, in a human clinical study of healthy control subjects, oral administration of H4 receptor antagonist significantly inhibited histamine induced itch compared to the placebo (Kollmeier et al., 2014). Clinical studies examining the effects of H4 agonists in AR subjects have not yet been reported. It is unclear if these novel H3 and H4 antihistamine compounds will ultimately prove effective as standalone treatments or as useful adjuncts to current treatments. It is also interesting to note that agents targeting neuro-sensory receptor pathways are being developed in the form of nasal sprays to help combat the neuronal component of AR and non-AR symptoms. Capsaicin is an example of a neuromodulatory agent, which is derived from red peppers. This compound is known to desensitize local sensory C-fibers and reduce nasal hyperresponsiveness, a key feature of AR and non-AR. In a randomized study of AR and non-AR subjects, intranasal capsaicin combined with eucalyptol used twice daily for 2 weeks significantly improved nasal congestion and sinus pressure/pain compared to the placebo (Bernstein et al., 2011). However, superior effect over the placebo for rhinorrhoea, sneezing or post nasal drip was not observed (Bernstein et al., 2011).

The concept of precision medicine, which has been recently reviewed elsewhere (Agache and Akdis, 2016; Muraro et al., 2016; Hellings et al., 2017), is a novel therapeutic approach which seeks to address the heterogeneity of disease and variability in response to treatment. The proposed key features of precision medicine include: personalized care based on molecular and immunologic endotyping of disease, patient participation in the decision-making process of therapeutic strategies, and consideration of the predictive and preventative component of the therapy. Biomarkers are measurable indicators that provide information about the pathophysiology of disease and response to treatment and are an integral component of endotype-driven precision medicine. In allergic diseases including AR, the development of biomarkers to predict and evaluate treatment response are complicated by vast heterogeneity, despite this some progress has been made. A specific HRH1 genotype was associated with treatment response to oral antihistamines in a cohort of Han Chinese subjects with AR (Chu, 2019). Although, the findings of this study should be confirmed in a larger cohort of subjects. Gu et al. (2017) identified that polymorphisms rs77485247 (TA+AA) and rs77041280 (TA+TT) within the histamine receptor 4 (HRH4) gene that were associated with reduced efficacy of oral H1 antihistamines and increased risk of adverse reactions in a cohort of AR patients. Given the HRH4 receptor is involved in the chemotaxis of mast cells and eosinophils, polymorphisms to this gene may be associated with worsened disease that requires more extensive treatment. It is estimated that 10–30% of patients with allergic disease or other autoimmune diseases experience a low or limited response to corticosteroids (Barnes and Adcock, 2009). To this effect, Wang et al. (2011) sought to identify proteins in nasal lavage fluid that could distinguish between AR subjects that were high responders or low responders to treatment with fluticasone nasal spray (50 μg twice daily). Proteins identified in nasal fluids, orosomucoid, fibrinogen alpha chain and apolipoprotein H were decreased significantly in the high responders and not the low responders, before and after corticosteroid treatment (Wang et al., 2011). Collectively, these findings demonstrate the utility of biomarker development in predicting response to treatment. Ultimately, the adoption of biomarker-based precision medicine in practice will improve management of disease and patient outcomes. Advancements in technology and more accessible tools for immunophenotyping including multiomic analysis, greater biobanking facilities and improved statistical tools to handle big data, will no doubt improve the ability to identify endotype specific biomarkers. In the future, we may see the one size fits all approach to management of AR symptoms replaced by endotype driven precision medicine.

## Conclusion

**FIGURE 6:**
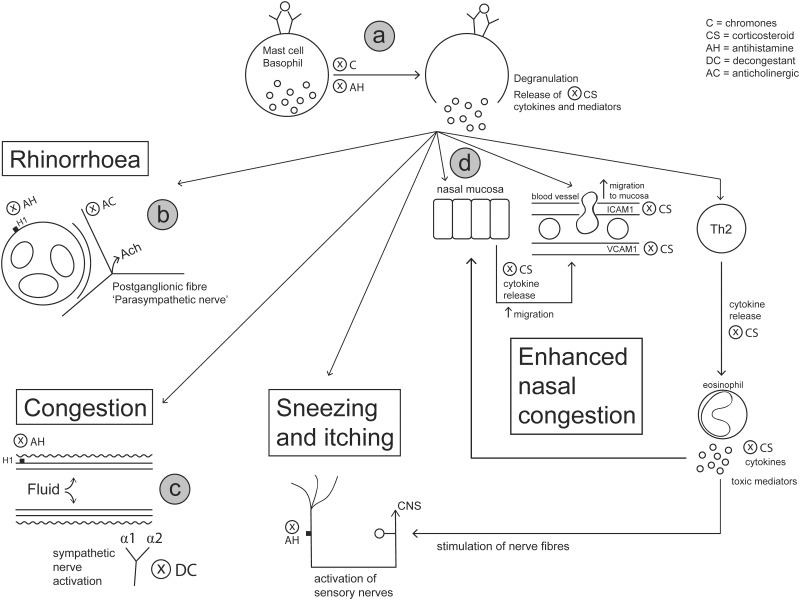
Intranasal medications prescribed for AR target different components of the allergic response to alleviate symptoms. **(a)** Antihistamines change the activity of histamine receptors to prevent the adverse effects of histamine on nerve endings, mucus glands and blood vessels. Stabilization of mast cells is provided by antihistamines and chromones, which prevent the degranulation of mast cells and downstream effects. **(b)** Anticholinergics prevent parasympathetic activation and secretion of mucus glands via antagonizing the action of acetylcholine on muscarinic receptors, thereby reducing the appearance of rhinorrhoea. **(c)** Decongestants activate adrenergic receptors which stimulate contraction of smooth muscles surrounding nasal vessels to prevent fluid leakage into tissues and reduce nasal congestion. **(d)** Corticosteroids act by modifying transcription of genes involved in allergic inflammation, thereby downregulating the production of cell signaling molecules and inhibiting the migration and activation of inflammatory cells. This action by corticosteroids limits the production of early phase symptoms (rhinorrhoea, sneezing and itching) and especially reduces nasal congestion associated with the late phase response.

A summary of the mechanisms of action of intranasal sprays for AR is presented in Figure 6. While many topical drugs have been developed to treat allergic inflammation and/or symptoms, there is no single drug available to target all components of the inflammatory process. Nasal decongestants and anticholinergics alleviate specific symptoms of AR such as nasal congestion and rhinorrhoea. Anthistamines and chromones act on specific inflammatory components of the allergic response, such as modifying the interaction of histamine with histamine receptors and preventing the release of histamine and other mediators from mast cells. Corticosteroids are the only class of drugs that posses a broad anti-inflammtory action. Corticosteroids exert their anti-inflammatory action by modifying multiple signal transduction pathways via transactivation and transrepression. These actions result in the downregulation of inflammatory cytokines, chemokines, mediators and cell adhesion molecules and also prevent migration of inflammatory effector cells to the nasal mucosa, collectively ameliorating key events underpinning AR symptoms. The future development of drugs to treat AR symptoms is confounded by the immense complexity of the disease pathophysiology. Despite this, new therapies for the treatment of AR are under investigation. The future of AR may include additional combination drug nasal sprays, the development of montelukast, H3/H4 receptor antagonists or capsaicin nasal sprays, and the adoption of endotype driven precision medicine.

## Author Contributions

AMW performed a critical review of the literature, drafted the manuscript text and prepared the figures and tables. NPW, contributed to the critical review of the literature and editing of the manuscript text. AWC and AJC contributed to the critical review of the literature, editing of the manuscript text, and review of figures.

## Conflict of Interest Statement

AWC, NW, and AJC have received funding from Mylan N.V. unrelated to the submitted work. The remaining author declares that the research was conducted in the absence of any commercial or financial relationships that could be construed as a potential conflict of interest.
